# Layer-by-Layer Fabrication of PAH/PAMAM/Nano-CaCO_3_ Composite Films and Characterization for Enhanced Biocompatibility

**DOI:** 10.1155/2022/6331465

**Published:** 2022-07-31

**Authors:** Naemi Tonateni Shifeta, Shindume Lomboleni Hamukwaya, Qi An, Huiying Hao, Melvin Mununuri Mashingaidze

**Affiliations:** ^1^School of Science, University of Namibia, Windhoek 12010, Namibia; ^2^School of Materials Science and Technology, China University of Geosciences, Beijing 100083, China; ^3^School of Engineering and the Built Environment, University of Namibia, Ongwediva 33004, Namibia

## Abstract

Nanoparticle production and functionalization for various biomedical uses are still challenging. Polymer composites constituted of poly(amidoamine) (PAMAM), polyallylamine hydrochloride (PAH), and calcium carbonate (CaCO_3_) nanoparticles have good biocompatibility with physiological tissue and fluids, making them excellent candidates for biomedical applications. This study investigated the characteristics of polymeric/nano-CaCO_3_ composite films based on a PAH/PAMAM matrix, which were fabricated through layer-by-layer synthesis on quartz glass substrates. It was found that the as-prepared elastic moduli of the resultant (PAH/PAMAM)_*n*_-CaCO_3_ (where *n* represents the number of bilayers) composite films varied from 1.40 to 23.70 GPa for different degrees of cross-linking when 0.1 M nano-CaCO_3_ particles were incorporated into the polymer matrix. The highly cross-linked (PAH/PAMAM)_15_-CaCO_3_ composite film had the highest recorded elastic modulus of 23.70 GPa, while it was observed that for all the composite films fabricated for the present study, the addition of the nano-CaCO_3_ particles approximately doubled the elastic modulus regardless of the degree of polymerization. Live/Dead assays were used to determine whether the produced composite films were compatible with human lung fibroblast cells. The findings indicate that the (PAH/PAMAM)_7.5_-CaCO_3_ composite film had the most positive effect on cell growth and proliferation, with the (PAH/PAMAM)_15_-CaCO_3_ composite film demonstrating significant ion transport behavior with low impedance, which was considered good for in vivo rapid cell-to-cell communication. Therefore, the (PAH/PAMAM)_7.5_-CaCO_3_ and (PAH/PAMAM)_15_-CaCO_3_ composite films are potential tissue engineering biomaterials, but further studies are essential to generate more data to evaluate the suitability of these composites for this and other biomedical functions.

## 1. Introduction

Over the past few years, bioinspired and biomimetic techniques have been used to generate functional materials that promote tissue engineering [1–3]. Compared with other surface synthesis methods, layer-by-layer (LbL) fabrication technology has garnered great interest due to its inherent simplicity, adaptability, and nanoscale control, among other characteristics. In recent years, there has been a great deal of research on LbL organic multilayer composite films that have been infiltrated with calcium carbonate [4, 5]. Various driving forces and assembly processes for LbL self-assembly have led to many LbL synthesized biomaterials, such as polyelectrolytes, biomolecules, and colloidal particles. These synthetic biomaterials have got physicochemical properties and functions that are useful in tissue engineering [6].

Biocompatibility is a significant issue when selecting or engineering biomaterials to circumvent adverse reactions in the host body, such as inflammation during tissue engineering [7–11]. Mechanical and structural properties are frequently required for tissue engineering to work well. Enhanced biocompatibility has become increasingly important in wound healing, bone repair, and spinal cord injury therapy [12–15]. Up to this point, significant effort has been directed toward enhancing the surface attributes such as stiffness, roughness, and hydrophilicity of LbL fabricated films [16]. Layer constituents cross-linking is the most extensively employed strategy among these techniques. Hillberg investigated the influence of genipin cross-linking on cell adhesion to LbL polyelectrolyte films using a cell linkage assay and discovered that cell adherence and spread on polymeric films were boosted by the cross-linking [17]. However, no comprehensive investigations on the film's surface chemistry and the respective impact on cell multiplication were conducted in the following years. Silva et al. [18] developed a method for adjusting cell adherence of LbL assembly films by using chitosan with or without cross-linking and coated with alginate. Cross-linking of multilayer films causes significant cell adhesion, spreading, and proliferation changes, which are linked to the alteration of surface chemistry and mechanical characteristics resulting from this process [18].

**Figure 1:**
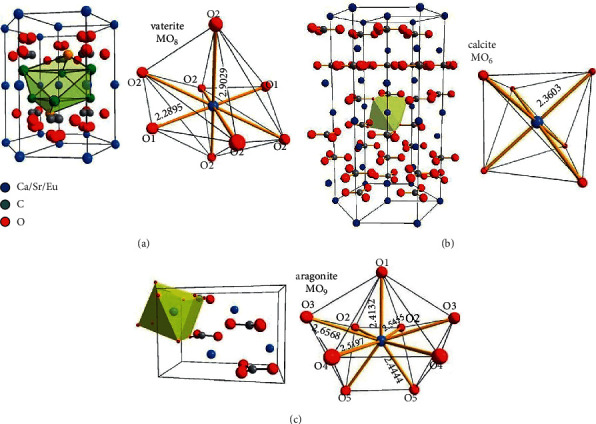
Allotropic structural cells of calcium carbonate: (a) vaterite, (b) calcite, and (c) aragonite. The neighboring environment of the cation and the cation-oxygen distances are also shown (copyright: Royal Society of Chemistry 2014, order number 1198792 [19]).

Coral shells and other organisms are rich sources of calcium carbonate, a vital biological mineral for life. Hydrated metastable forms of calcium carbonate exist in monohydrocalcite (the most stable form), calcium carbonate hexahydrate (the second most stable form), and the unstable amorphous calcium carbonate (ACC) phases. These phases are known by their mineral names, which are calcite, aragonite, and vaterite [19, 20], respectively, as shown in Figure 1. When water is dissolved in the vaterite polymorph, it forms nonporous calcite (the most stable polymorph). CaCO_3_ mineralization and biomimetic synthesis have attracted research interest due to their prevalent use in various industries. In recent years, the biocompatibility and biodegradability of CaCO_3_ in pharmaceutical and biological applications have been extensively studied [21, 22]. Several studies have enhanced the mechanical properties of polymeric/nano-CaCO_3_ [23–27], with the underlying variables being the film architecture and fabrication methods employed.

A poly (amidoamine) (PAMAM) dendrimer acts as a hydrophobic micro-container for encapsulating small molecules and nanoparticles in hydrophilic environments via electrostatic or hydrophobic interactions [5–9]. PAMAM has been studied for retarded release in LbL multilayers [10]. Most earlier studies created LbL multilayers utilizing noncovalent interaction, which may result in insufficient interfacial bond strength. The influence of covalent cross-linking on retarded release in LbL PAMAM multilayers needs additional study [10, 15]. This study combined covalent interlayer connections with drug reservoirs to retain model drug molecules. Multilayers and the drug reservoir are made from PAMAM dendrimers with carboxylic terminal groups. First, standard LbL multilayers are made, and then post-infiltration is followed by photochemical cross-linking to form covalent interlayer links.

In numerous labs, poly (allylamine hydrochloride) (PAH) has been examined separately and as a combination of polyelectrolyte connecting mechanisms, including the usage of PAMAM dendrimers [10]. Researchers employed concentrations, molecular weight, and ionic strength to modify the film thickness of polymeric multilayer films generated by successive self-assembly of aqueous nanoparticles with PAH and poly (ethyleneimine).

PAMAM dendrimers are widely employed as scaffolds for biomedical applications, particularly in tissue engineering and drug delivery systems, due to their good biocompatibility, biodegradability to harmless chemicals, and bioactivity [28]. Despite their high biocompatibility, these biopolymers are challenging to manufacture and have poor mechanical characteristics [29]. In this work, PAH/PAMAM/nano-CaCO_3_ composite films were successfully synthesized, characterized, and then evaluated using Live/Dead assays for enhanced biocompatibility with human lung fibroblast cells (HLFCs). HLFCs are isolated from fully developed lung tissue and are the most prevalent type of cell in lung interstices. HLFCs are important for controlling inflammation and repair of pulmonary tissue, making these cells very useful in studying and treating respiratory diseases or disorders like asthma, tuberculosis, and lung cancer, and for pulmonary wound repair.

## 2. Materials and Methods

The following reagents were used as supplied and procured from Sinopharm Chemical Reagent Beijing Co., Ltd.: NaOH, CaCl_2_, Na_2_CO_3_, poly(allylamine hydrochloride), and PAH (Mw, 15000). Sigma-Aldrich supplied the ethylenediamine core, generation 3.5 solution (PAMAM dendrimer) (Mw, 12927.67), and methylene blue. TCI provided the disodium of 4,4′-dialzide-2,2′-stilbenedi-sulfonic acid (DAS). The full list of chemical reagents used in the study is given in Table 1. A commercially 400 W multiwavelength high-pressure mercury lamp with a 300–400 nm light wavelength was used to perform photochemical cross-linking.

### 2.1. Synthesis of the Piezoelectric Composite Film Polyvinylidene Fluoride-Hexafluoropropylene (PVDF-HFP) Solution

The PVDF-HFP solution was made by dissolving 1 g PVDF-HFP and 1 g PVDF-HFP copolymer in 7 mL dimethylformamide (DMF) and placed in the oven for 1.5 h at 80°C. The solution was annealed on a freshly cleaned glass plate, and the necessary thickness was achieved by spreading the film on a glass stick to control the thickness and allowing it to cool at room temperature. DMF was evaporated by placing the substrate in an oven for 6 h at 80°C. The resultant films were cut into 3 cm × 3 cm squares and placed in plasma cleaner to improve the membranes' hydrophilicity. The PVDF-HFP membrane was used for the LbL method of PAH/PAMAM_15_, which was followed by the biomineralization of CaCO_3_.

### 2.2. Fabrication of Cross-Linked PAH/PAMAM Multilayers on Quartz Glass/Indium Tin Oxide (ITO) Substrates and Piezoelectric Film

The piezoelectric film substrates were first cleaned for 10 min with a plasma cleaner for hydrophilicity. The LbL PAH/PAMAM multilayers were then assembled as previously described by [28–30]. To obtain the low, medium, and high cross-linking multilayers, the (PAH/PAMAM)_*n*_ as-prepared multilayers sample were submerged in 5 mg·mL^−1^ (pH 3.8) of DAS aqueous solution for 10 s, 3 min, and 40 min (note that the subscript *n* denotes the number of bilayers whereby each substitute represents 0.5 layers). Noncross-linking samples were not immersed in DAS and were used as a control. The multilayer film substrates were illuminated for 5 min with a 400 W mercury lamp at a distance of 20 cm after being dried by nitrogen flow. To test the stability of the composite film multilayers, the substrates were submerged into an aqueous NaOH solution (pH 12) for 3 min after photochemical cross-linking to assess the multilayer stability. Both quartz and ITO glass were used as substrates for multilayer preparation and processed similarly.

### 2.3. Mineralization of Calcium Carbonate Nanoparticles in the LbL PAH/PAMAM Matrices

The multilayered films were immersed in 0.1 M CaCl_2_ solution for 30 min before being immersed in 0.1 M Na_2_CO_3_ solution for a further 30 min to produce CaCO_3_ nanoparticles and then air-dried at ambient temperature; this method was adopted from our previous work [30, 31].

### 2.4. Sample Preparation Procedure

**Scheme 1:**
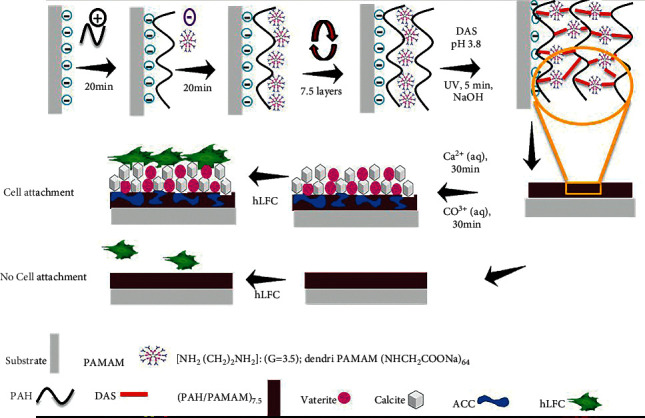
Schematic diagram of LbL fabrication process of cross-linked (PAH/PAMAM)_7.5_ multilayers, biomineralization of CaCO_3_, and cell culture using HLFC.

The polyelectrolytes PAH (pH 9.5) and PAMAM (pH 7) with carboxyl-terminal groups were utilized as a prototype for incorporating multilayer film in a layer-by-layer method to demonstrate the methodology (Scheme 1). A previous method of photochemical cross-linking was used to produce the multilayer film samples [29, 30], and the researchers believe that hydrogen bonding and electrostatic interactions were the driving forces behind multilayer synthesis.

### 2.5. Characterization

Table 2 lists the major instruments used in this study for materials characterization purposes.

#### 2.5.1. Physiochemical Properties

A Dimension 3100 atomic force microscope (AFM) obtained from Veeco, USA, was used to analyze the polyelectrolyte multilayers' structure and surface morphology under a Tapping Mode probe with constant amplitude. Peak Mode QNM tapping was used to measure the nanoscopic Elastic's moduli on the AFM. The AFM images of the nanocomposites samples were then used for digital scan image processing with the commercial software Sigma Scan Pro (Sigma Co., US). The surface morphology of calcium carbonate and the multilayer morphology were both studied using a scanning electron microscope (SEM), JSM 7401. D/MAX-RC diffractometer (Rigaku, Japan) patterns of powder X-ray diffraction (XRD) were acquired at an 8°/min scanning rate (40 kV, 100 mA) to analyze the crystal structure of the prepared composite film materials, identify the crystalline phases present in a composite material, and reveal information about its chemical composition.

The Raman spectroscopy and mapping were conducted on a Micro-Raman spectrometer (Horiba), mapping area = 5 *μ*m × 5 *μ*m, to generate detailed chemical images based on a sample's Raman spectrum. An electrochemical workstation was used to measure the electric potential (PGSTAT 302 N, Metrohm Autolab B. V.). Ahead of measurement, silver paint (Agar No. 0443) was used to engineer silver electrodes on all sides of the composite film substrates to ensure precise electrode cutting from the samples.

#### 2.5.2. Electrical Characteristics

A CHI760 E electrochemical workstation was used to perform and measure the cyclic voltammetry scanning (CV) and electrochemical impedance (EIS) (Chenhua, Shanghai, China) of the nanocomposites. The studies were carried out in a three-electrode configuration with phosphate-buffered saline (PBS) pH 7.4, 0.1 M as the backup electrolyte. The multilayers built on the ITO-glass substrate served as the working electrodes. At the same time, the measurements with a platinum counter electrode in addition to an Ag/AgCl reference electrode were utilized. Different electrical potentials of 5 mV amplitude were applied to the electrochemical workstation for the EIS measurements.

#### 2.5.3. Molecular Adsorption and Release

Methylene blue (MB) at pH 9, 0.1 M and gentamycin sulfate (pH 7, 0.1 M) aqueous solutions were used as adsorbate solutions for all four (PAH/PAMAM)_7.5_-nano-CaCO_3_ composite film samples, namely, noncross-linked, low cross-linked, medium cross-linked, and highly cross-linked. It took 60 min for the adsorbates to attain equilibrium concentration on the composites. In order to eliminate molecules that were only superficially adsorbed during the loading process, all-composite film samples were briefly washed in deionized water before determining the absorbance of the multilayer films. Thereafter, the films were submerged in PBS solution in order to conduct the molecular release trials and pressed during this release process to induce piezoelectricity. A specified time interval was chosen to measure the absorbance of the release solution by taking 6 mL of the solution and returning it after the measurement. (PAH/PAMAM)_7.5_ and (PAH/PAMAM)_7.5_-nano-CaCO_3_ medium cross-linked composite film samples were further subjected to molecular desorption tests using the same test procedures but only at pH 5.4 and 7.4 to determine the impact of the calcium carbonate mineral on the desorption characteristics of the composite films.

#### 2.5.4. Cell Culture

A Zeiss Leica-440 inverted reflectance laser scanning confocal microscope (Zeiss, Jena, Germany) was used to examine cell morphology. HLFC layers were grown on high-glucose Dulbecco's modified eagle medium (DMEM) containing 10% fetal bovine Serum and an antibiotic-antimycotic solution comprising 100 *μ*gL^−1^ penicillin and streptomycin sulfate as previously described by [31]. The culture cells were grown in a moistened atmosphere with 5% CO_2_ and 95% air at 37°C. Quartz glass 1 cm × 1 cm square quartz glass-LbL films were disinfected for 30 min using a Co 60 (*γ*) laser and then inserted in a 24-well plate cavity for subsequent cell seeding. The cultivated cell density of the samples was 1 × 105 per well. After incubation for 24 h, cells were stained for 10 min through the LIVE/DEAD Viability/Cytotoxicity Kit, then rinsed three times using PBS solution to eradicate free methylene dye before observing cell morphologies. In addition to the as-prepared composite nanoparticle test, the LIVE/DEAD assay determines cellular membrane stability and cytoplasmic enzyme performance. The assay's effectiveness is predicated on the capability of healthy cells.

## 3. Results and Discussion

### 3.1. Morphological Structure by SEM and Transmission Electron Microscopy (TEM)

**Figure 2:**
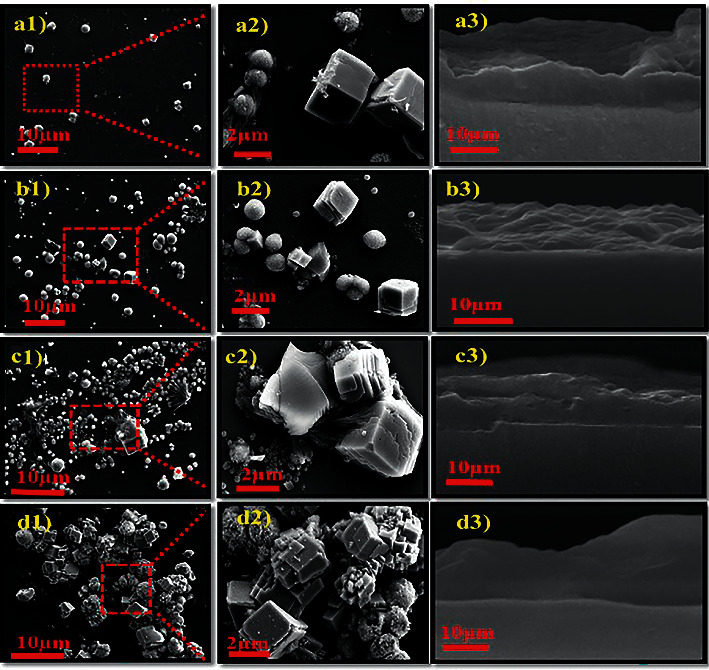
SEM images of calcium carbonate of (a1–a3) noncross-linked, (b1–b3) low cross-linked, (c1–c3) medium cross-linked, and (d1–d3) highly cross-linked (PAH/PAMAM)_7.5_ multilayers.

**Figure 3:**
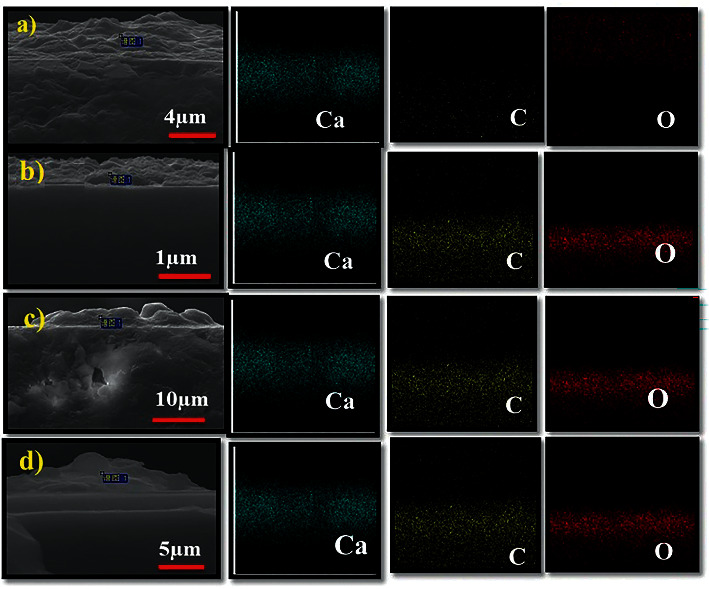
SEM and EDX analysis of (PAH/PAMAM)_7.5_-CaCO_3_ composite. Elemental mapping of calcium, carbon, and oxygen from all the components on the cross section multilayers of (a) noncross-linked, (b) low cross-linked, (c) medium cross-linked, and (d) highly cross-linked films.

Electron micrographs of PAH/PAMAM)_7.5_-CaCO_3_ nanocomposites were obtained from (Figures 2(a)–2(d)) and 3(a)–3(d)).  Figure 2, with all cross-linking degrees, shows similar polymorphs of CaCO_3_ with spherical and rhombohedral shapes embedded in the film. The results show the characteristics of columnar crystals of spherical vaterite and rhombohedral calcite [28, 32–34]. The SEM results clearly show that as the degree of cross-linking increases, the shape of the crystals changes. The small smooth vaterite crystals are transformed into rough, larger, more porous, irregular spherical aggregates, as shown in Figure 2.

On the other hand, calcite's smooth well-faceted rhombohedral morphology was transformed into larger irregular aggregates with rough faces. This is because there was nucleation on the existing crystal. Therefore, the nanocomposite morphology was greatly influenced by cross-linking degrees, as shown in SEM-EDX analysis (Figures 3(a)–3(d)) and the S1 morphological characterization of CaCO_3_ on the PVDF-HFP membrane. Elemental mapping of calcium, carbon, and oxygen was detected from all components on the cross section multilayers as shown in S1(c) and S1(d).

**Figure 4:**
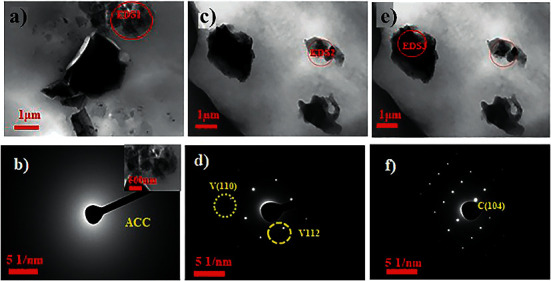
TEM and SAED of (a, b) amorphous calcium carbonate, (c, d) vaterite, and (e, f) calcite obtained from the medium cross-linked of (PAH/PAMAM)_7.5_-CaCO_3_ from PVDF-HFP substrate.

The crystallographic direction of the nanocrystals was examined with a TEM (Figures 4(a), 4(c), and 4(e)) and selected area electron diffraction (SAED) analysis (Figures 4(b), 4(d), and 4(f)). The TEM and SAED images (Figures 4(a) and 4(b)) attest that an ACC structure was dispersed within the matrix. The spherical with irregular, rough surface structures are confirmed to be vaterite, and the irregular are rhombohedral calcites. The SAED images (Figures 4(d) and 4(f)) show single crystals of calcite and vaterite, respectively.

### 3.2. XRD and FT-IR

**Figure 5:**
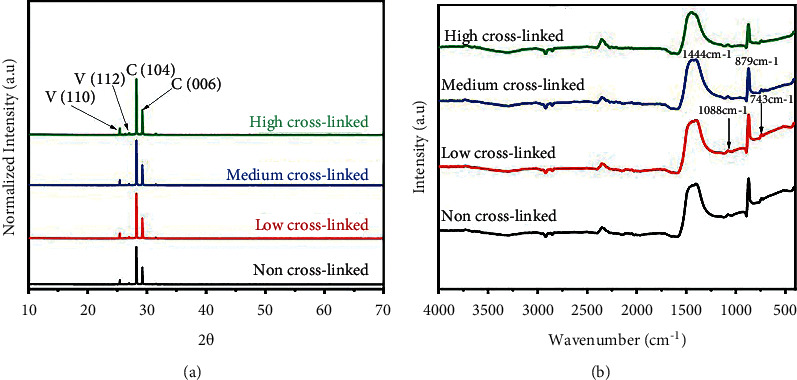
(a) XRD patterns and (b) FT-IR obtained from all cross-linked samples of PAH/PAMAM_7.5_-CaCO_3_ on a quartz glass substrate.

The X-ray diffraction measurements (Figure 5(a)) showed that the obtained crystals are all mixtures of calcite and a small amount of vaterite. The XRD patterns of the nanocomposite films exhibited high-intensity peaks at 2*θ* values at 28.1°, and 29.4° corresponds to (104, 006) planes in the calcite (JCPDS no. 88-1807) [35]. The 2*θ* values at 25.4° correspond to (112) plane in the vaterite according to (JCPDS no. 33-0268) to vaterite [36, 37]. There is a small peak at 2*θ* values 27° corresponding to vaterite.

In the mid-FT-IR spectrum (examined from 4000–500 cm^−1^), the absorption bands of amorphous calcium carbonate (carbonate ion) can be split into four regions: *v*1 = 1080 cm^−1^, *v*2 = 870 cm^−1^, *v*3 = 1400 cm^−1^, and *v*4 = 700 cm^−1^. According to FT-IR (Figure 5(b)), all cross-linked samples of PAH/PAMAM7.5-CaCO_3_ on a glass quartz substrate have the main peaks at 1444 cm^−1^ (*v*3) and 879 cm^−1^ (*v*2), which are characteristic absorptions of calcite, while the symmetric stretches of 1088 cm^−1^ (*v*1) and 743 cm^−1^ (*v*4) are characteristic absorptions of vaterite assigned to carbonates (CO_3_^2-^) from calcium carbonate crystals. Similar findings have previously been observed for calcium carbonate crystals [27, 33, 34]. The stretching vibrations of C-H groups in the PAH/PAMAM multilayers cause the distinctive peaks to shift. The formation of hydrogen bonds between the C-H group in PAH and the C=O functional group in PAMAM was linked to the redshift of the C-H bond, which resulted in a drop in the electron cloud density of the C-H bond. The redshifts of N-H and C=O were supposed to be caused by charge transfer and hydrogen bonding between PAH and PAMAM [38]. Kim and Park reported that the presence of C-N stretching (amine) at 1088 cm^−1^ in a composite sample showed that polymerization happened early in the CaCO_3_ mineralization [39]. The intensities of these peaks improved as the number of bilayers increased from 0 to 7, demonstrating the successful production of the PAH/PAMAM multilayers.

### 3.3. Raman Spectroscopy and Mapping

**Figure 6:**
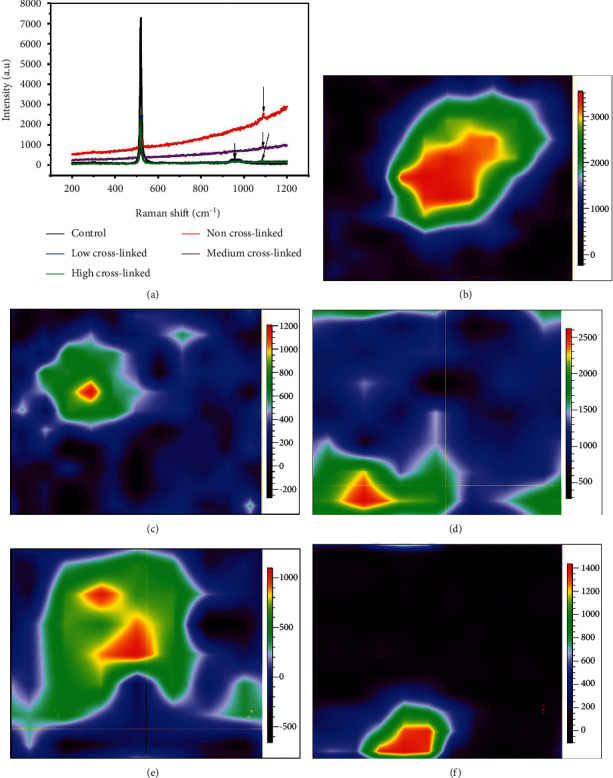
(a) Raman spectrum of all cross-linkages and Raman mapping of (b) noncross-linked (c) low cross-linked, (d) medium cross-linked, and (e) highly cross-linked (PAH/PAMAM)_7.5_-CaCO_3_. (f) PAH/PAMAM)_7.5_ -CO_3_^2−^ (control) (dimension of mapping area = 5 *μ*m × 5 *μ*m).

In Figure 6(a), the Raman spectra show a sharp peak at 520 nm for all the film samples under consideration. This is the laser excitation line for a quartz glass substrate, which was used as the substrate material to fabricate all the samples for this study. Additional peaks are located between 1085 cm^−1^ and 1087 cm^−1^. The peak at 1085 cm^−1^ for the cross-linked composite film suggests the formation of ACC, and the peak at 1087 cm^−1^ is assigned to carbonates *v*1 symmetric stretch modes of calcite and vaterite, respectively [26]. The film was only immersed in Na_2_CO_3_ solution for the control sample, and thus the results only show a broad peak at 950 cm^−1^, with no peak for carbonate observed. Figures 6(b)–6(f)) illustrate the results of the Raman color mapping for noncross-linked composite films, which show a fair intensity with a maximum peak of 1087 cm^−1^ Raman peak (*v*1 CO_3_) [40]. The CO_3_^2−^ content mapping results illustrate the history of the carbonation process and the development of carbonation in specific areas. Carbonation appears to have begun at the exposed surface (blue area), with the carbonation front steadily increasing with time.

### 3.4. AFM

**Figure 7:**
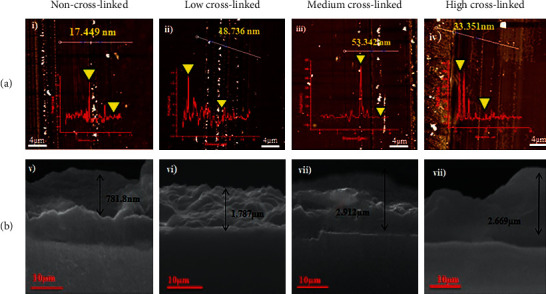
Film thickness representation before and after mineralization. (a) AFM of film thickness before mineralization of (PAH/PAMAM)_7.5_ and (b) SEM cross section of the films after mineralization of (PAH/PAMAM)_7.5_ -CaCO_3_.

Cross-linked (PAH/PAMAM)_7.5_ multilayers film thickness data (Figures 7(a)(i)–7(a)(iv)) show thickness measurements of 17.449 nm, 18.736 nm, 53.342 nm, and 33.351 nm for non, low, medium, and high cross-linking films, respectively. The stability of the multilayers during the fabrication of cross-linked PAH/PAMAM multilayers confirmed the significant variation in film thickness before mineralization. After immersion of the PAH/PAMAM multilayers in the basic solution (NaOH) for 3 min, noncrossed-linked films disintegrated completely, resulting in low film thickness compared to other prepared films [30]. After mineralization, the four-film samples were not smooth. Hence, AFM could not measure the thickness of the multilayer films, and SEM was therefore used to measure the thickness after the growth of calcium carbonate. The results (Figures 7(b)(v)–7(b)(viii)) show a drastic increase in the film of 781.8 nm, 1.787 *μ*m, and 2.912 *μ*m, 2.669 *μ*m for non-, low-, medium-, and highly cross-linked composite films, respectively. The fully crossed-linked film exhibited a decrease in film thickness as previously reported [30] because the film became more compact, leading to confined spaces within the multilayer structure [41].

### 3.5. Mechanical Properties

Quartz glass substrates were used to prepare all samples for characterization with the AFM. The peak force tapping AFM was applied to probe the nano-indentation of (PAH/PAMAM)_7.5_ to determine the elastic modulus before and after mineralization. Elastic modulus data in Table 3 show that (PAH/PAMAM)_7.5_ film and(PAH/PAMAM)_7.5_-nano-CaCO_3_ film stiffnesses rise by 10 and 17 times, respectively, with an increasing degree of cross-linking. Before mineralization, the average elastic modulus for (PAH/PAMAM)_7.5_ was 5.2 GPa, and after mineralization, the average elastic modulus (PAH/PAMAM)_7.5_-CaCO_3_ was determined to be 10.6 GPa. As a result, the modulus increases by nearly 2 times. The results demonstrate a significant improvement in mechanical characteristics and are equivalent to natural materials such as bones and nacres. Unmineralized bone collagen has a modulus of nearly 1–2 GPa, which increases to 10–20 GPa during mineralization [5].

In vivo, HLFCs experience stress and strain due to the rhythmic nature of the respiratory cycle, thus certain mechanical properties are important for their optimal function. Tensile strength and elastic modulus are good indicators of whether a synthesized biomaterial can perform to expectations under stress-strain conditions in vivo. The elastic moduli for tissue culture plastic and glass are in the 2 to 4 GPa range [42], validating the stiffness achieved for the composites in this study, factors such as composition and degree of mineralization as determinants of the elastic modulus of different body tissues and organs. HLFCs in vivo are likely to experience an elastic modulus in the 1 kPa range, meaning these composites should be expected to perform mechanically for pulmonary functions, but because of the extreme stiffness for the cross-linked samples, they are probably best suited for other tissue engineering applications where high stiffness and strength are required [43]. But the volume fraction of the nano-CaCO_3_ and the degree of polymerization can be regulated in a series of experiments until the desired elastic moduli and strength are obtained.

### 3.6. Piezoelectric Properties

**Figure 8:**
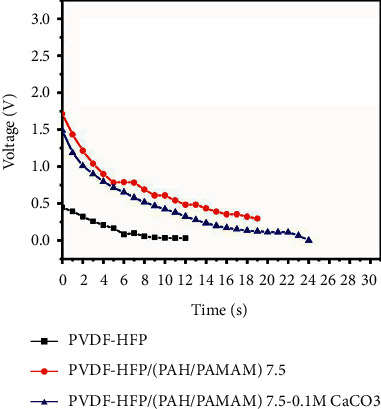
Output voltage-time curve of PVDF-HFP, PVDF-HFP/(PAH/PAMAM)_7.5_, and PVDF-FP/(PAH/PAMAM)_7.5_-CaCO_3_ after finger-pressing.

The piezoelectric capabilities of the prepared films were investigated by creating electric signals by pushing the film with a finger to generate the piezoelectric effect, which caused film deformation and resulted in combined piezoelectric and dielectric production [31]. PVDF-HFP/(PAH/PAMAM)_7.5_ generated a voltage of 1.75 V in response to the finger push force of 5 N, which decreased to 0 V after 19 s, as shown in Figure 8. In comparison, pure PVDF-HFP produced an electric potential of 0.5 V, which dropped to 0 V after 12 s. PVDF-HFP/PAH/PAMAM)_7.5_-CaCO_3_ nanocomposite film, on the other hand, developed a voltage of 1.5 V that decreased to 0 V after just 25 s.

### 3.7. Electrochemical Properties: Impedance and Cyclic Voltage

**Figure 9:**
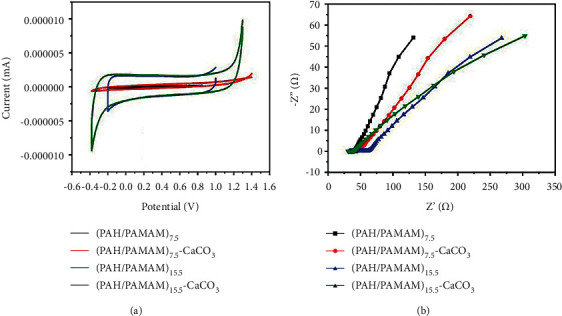
Electrical properties (a), cyclic voltammograms, and (b) Nyquist plot (at a scan rate of 5 mVs^−1^) of different films in 0.1 M PBS.

The electrical characteristics of medium cross-linked polymeric/nano-CaCO_3_ films coated on ITO [44] were examined using CV scanning (Figure 9(a)) in the presence of Ag/AgCl in 0.1 M 0.1 M PBS, pH 7.4. The films with (PAH/PAMAM)_7.5_ exhibited limited electrical responsiveness. In contrast, the anodic and cathodic currents markedly increased for the composite film samples of (PAH/PAMAM)_7.5_-CaCO_3_, (PAH/PAMAM)_15.5_, and (PAH/PAMAM)_15.5_-CaCO_3_, which attributed to the porous structures that facilitate fast-moving electron charge transfer. The composite film with the highest current response was (PAH/PAMAM)_15.5_-CaCO_3_. This suggests that the (PAH/PAMAM)_15.5_-CaCO_3_ is more conductive in PBS than the other three composite films. EIS in PBS was used to examine further the conductivity and electro-activity of the polymeric/nano-CaCO_3_ layer.

In the high-frequency range, the Nyquist plots for both (PAH/PAMAM)_15.5_, (PAH/PAMAM)_7.5_-CaCO_3,_ and (PAH/PAMAM)_15.5_-CaCO_3_ films revealed a quasi-semicircular arc, indicating a strong redox activity (Figure 9(b)). The (PAH/PAMAM)_15.5_-CaCO_3_ composite film samples demonstrated significant ion transport behavior with low impedance. The diameter of the semicircles matches with the RCT, supporting the CV data. The low electrical resistance is advantageous for in vivo rapid cell-to-cell transmission using spontaneous bioelectrical signals generated by ion and pump activity among nearby cells [45].

### 3.8. Molecular Adsorption and Release

**Figure 10:**
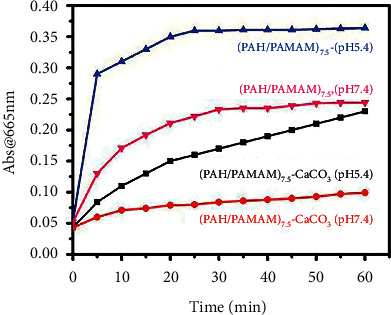
The release profile of MB from the medium crossed-linked (PAH/PAMAM)_7.5_ film and (PAH/PAMAM)_7.5_-CaCO_3_ film at various pH values.

The adsorption of protein molecules on the surfaces of biomaterials is an indispensable feature of the chemical and physiological reactions at the biomaterial and biological environment interface. Synthetic biomaterials implanted in the living body are expected to be able to release different functional molecules for protein signaling to nearby tissues or fluids [46]. Thus, evaluation of the biomaterials/proteins interaction and molecular adsorption/desorption of proteins on synthetic biomaterial surfaces is critical for many biomedical functions, such as drug delivery and tissue engineering [47].  Figure 10 illustrates the impact of the CaCO_3_ nanoparticles on MB release from the medium cross-linked (PAH/PAMAM)_7.5_-nano-CaCO_3_ and (PAH/PAMAM)_7.5_ composite films at pH 5.4 and 7.4. The CaCO_3_ nanoparticles lower the MB desorption at either pH, but pH 5.4 consistently results in much improved molecular release, as shown in Figure 10. Thus, the absence of the mineral and low pH promotes MB release. The results show that MB was completely released during the first 30 min at both pH values for either film before mineralization, whereas the release was sustained after mineralization. The graphs in S2 show that increasing the degree of cross-linking increases the MB adsorption capacity of the (PAH/PAMAM)_7.5_-nano-CaCO_3_ composite film, while the optimum pH for MB molecular desorption is 4.5. Antibiotic drugs and gentamycin sulfate were added to the calcium carbonate-loaded multilayers. Therefore, S3 illustrates that low pH and a high degree of cross-linking favor adsorption and desorption of gentamycin sulfate molecules from the (PAH/PAMAM)_7.5_-nano-CaCO_3_ composite films. The release of GS in PBS (pH 7.4 and 5.4) from calcium carbonate-loaded multilayers is also pH-dependent, as shown in S3. This was due to the pH-dependent solubility of CaCO_3_, which causes the matrix to disintegrate in an acidic environment, releasing most of the adsorbed drugs [48]. However, since the pH of human blood is generally in the 7.35–7.45 range, the (PAH/PAMAM)_7.5_ film can be considered ideal for fast in vivo drug delivery applications. Still, if retarded drug delivery is the goal, then the (PAH/PAMAM)_7.5_-nano-CaCO_3_ film is the better biomaterial.

### 3.9. Cell Culture

**Figure 11:**
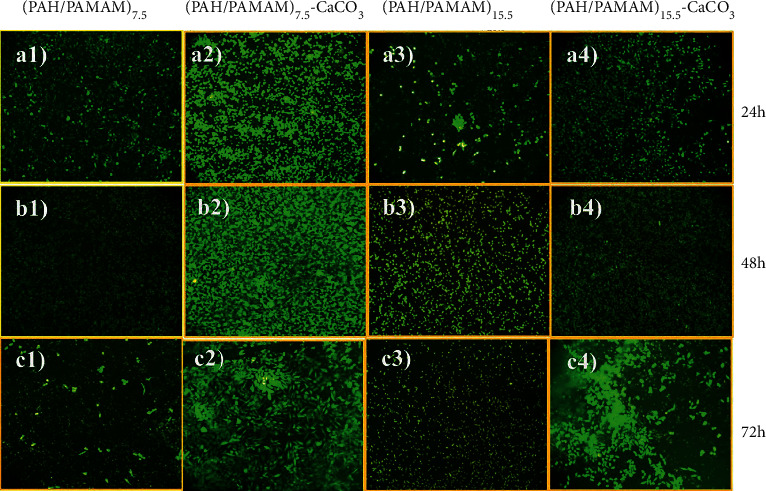
Fluorescent images of HLFCs cultured (a1–a4) 24 h, (b1–b4) 48 h, and (c1–c4) 72 h on substrates with different bilayers. Control experiment, no CaCO_3_.

The medium cross-linked composites films were used to test the biocompatibility for cell culture of HLFCs using LIVE/DEAD assays. Two parameters were considered: the organic content and the presence/absence of CaCO_3_. Composites with 7.5 and 15.5 multilayers of PAH/PAMAM but no CaCO_3_ served as substrates for the control experiments. Films with CaCO_3_ were used as test experiments, and as shown in the early stage of the 24-cell culture, the cells seeded onto the four samples attached well to the substrates and showed elongated morphology, which corresponds with healthy features of the fibroblast cells (Figures 11(a1)–11(a4)). However, as shown in Figure 11(a2), the number of cells on (PAH/PAMAM)_7.5_-CaCO_3_ films was higher than that of the three other samples. After 72 h, the difference in cell morphology and cell density was even clearly distinct. The cells on (PAH/PAMAM)_7.5_-CaCO_3_ remained healthy and exhibited elongated shapes and increased cell density, illustrating the effect of cell proliferation, as shown in Figures 11(b2) and 11(c2), while the cells on the other films exhibited shrunken round morphology with irregular aggregates [49] as well as a decrease in cell density due to dead cells that were no longer attached to the substrate Figures 11(b1), 11(c1), 11(b3), 11(c3), 11(b4), and 11(c4). Nevertheless, both film composites were biocompatible and favored cell growth and proliferation.

Furthermore, the morphology of the cells on the (PAH/PAMAM)_7.5_-CaCO_3_ was more elongated and stretched than the other films because of the positive effect of the organic-inorganic content. The hard film, the film with low organic matter content, favors cell attachment and proliferation over softer films with high organic content (PAH/PAMAM)_15.5_-CaCO_3_. Because of the little toxicity and high-tissue compatibility [28], the results show that this polymeric/nano-CaCO_3_ film can potentially be used for tissue engineering.

## 4. Conclusions

The LbL method was used to fabricate bioinspired, mechanically tough, and biocompatible polymeric (PAH/PAMAM)_*n*_ films, some with and others without nano-CaCO_3_ particles. The stiffness of the (PAH/PAMAM)_7.5_ film and the (PAH/PAMAM)_7.5_-nano-CaCO_3_ film rose by 10 and 17 times, respectively, with an increasing degree of cross-linking from noncross-linked to fully cross-linked, and with the addition of nano-CaCO_3_ particles. Fluorescent images showed increased proliferation of HLFCs, especially for the PAH/(PAMAM)_7.5_-CaCO_3_ films. (PAH/PAMAM)_15.5_, (PAH/PAMAM)_7.5_-CaCO_3,_ and (PAH/PAMAM)_15.5_-CaCO_3_ films exhibited strong redox activity, while the highest current response was observed for (PAH/PAMAM)_15.5_-CaCO_3_ composite film sample. In the biocompatibility test, the cells on (PAH/PAMAM)_7.5_-CaCO_3_ remained healthier and exhibited better-elongated shapes and increased cell density compared to the (PAH/PAMAM)_15.5_-CaCO_3_ composite film sample. Thus, lower polymeric content in the composite films favors cell attachment and proliferation but reduces the current responsiveness. The presence of nano-CaCO_3_ particles in the synthetic composite films had a negative effect on molecular adsorption and release, implying potential unsatisfactory performance in drug delivery applications. Overall, the results suggest that the composite films have a potential application in tissue engineering to stimulate cell growth and proliferation. Furthermore, the nano-CaCO_3_ particles can be utilized as filling agents in organic multilayered films to improve their mechanical properties for biomedical functions.

### 4.1. Future Work

The synthesized nanomaterial composites have high tissue compatibility and biomineralization, which could be exploited for biomedical functions in the future. The slow molecular release kinetics can be exploited for controlled application of medication in certain circumstances or to avoid squandering expensive treatments by optimizing the release of treatment from a matrix. To better understand how LbL nanoparticles interact with biological systems, further studies are needed to investigate factors like strength, elasticity, and shape and how these influence biological responses in tissue engineering. Extensive biocompatibility tests over prolonged periods are required in order to determine the suitability of these composites for biomedical applications. Computer-aided nanomaterial preparation is crucial to the reproducibility and speed of preparation of tissue engineering upgrades. Thus, the application of Computer-Aided Design (CAD) systems in the design and modeling of the composites is recommended, while other specialized modeling systems can be deployed to study their in vivo behavior.

## Figures and Tables

**Table 1:** Composition of reagents used.

Reagents	Concentration
NaOH (aq)	0.1 M
CaCl_2_ (aq)	0.1 M
Na_2_CO_3_ (aq)	0.1 M
Poly (allylamine hydrochloride)—PAH	0.1 M
PAMAM dendrimer	0.1 M
Disodium of 4,4'-dialzide-2,2'-stilbenedi-sulfonic acid (DAS)	0.1 M
Dimethylformamide (DMF)	0.1 M
Methylene blue	0.1 M
Phosphate-buffered saline (PBS) solution	0.1 M
Gentamycin sulfate	0.1 M
Dulbecco's modified eagle medium (DMEM)	4.5 g/L glucose + 10% fetal bovine serum + 100 *μ*g/L penicillin and streptomycin sulfate + amino acids + vitamins + inorganic salts + 15.9 mg/L phenol red·Na

**Table 2:** Instruments used for characterization of the synthetic nanocomposites.

Instrument	Evaluation function
Dimension 3100 atomic force microscope	Film structure and surface morphology
Scanning electron microscope (SEM)	Surface morphology of calcium carbonate nanoparticles and composite films
D/MAX-RC diffractometer (Rigaku, Japan)	Identification of the crystal structure, crystalline phases present in the composites, and the chemical composition
Micro-Raman spectrometer (Horiba)	CaCO_3_ mineralization in the composites and spatial distribution
CHI760 E electrochemical workstation	Cyclic voltammetry scanning (CV) and electrochemical impedance (EIS)
Zeiss Leica inverted reflectance laser scanning confocal microscope	Cell morphology

**Table 3:** Elastic moduli (*E*) of the Si substrate and synthesized (PAH/PAMAM)_7.5_ films before and after mineralization.

	Film stiffness, *E* (GPa)				
	Substrate	Noncross-linked	Low cross-linked	Medium cross-linked	Highly cross-linked
Before nano-CaCO_3_ addition	0.11	0.99	3.50	6.22	10.00
After nano-CaCO_3_ addition	—	1.40	6.02	11.20	23.70

## Data Availability

On request, the data used to support the findings of this study can be obtained from the corresponding author.

## Supplementary Materials

Supplementary Materials Supplementary materials S1: SEM and TEM morphological characterizations of CaCO_3_ on the PVDF-HFP membrane and the corresponding EDS spectra with elemental composition. S2: performance characterization loading and release of a cationic dye (MB) into multilayers of (PAH/PAMAM)_7.5_-CaCO_3_ with different cross-linking degrees. S3: performance characterization loading and release of a cationic antibiotic (GS) into multilayers of (PAH/PAMAM)_7.5_-CaCO_3_ with different cross-linking degrees.
