# Supplementary material for: Layer-by-Layer Fabrication of PAH/PAMAM/Nano-CaCO3 Composite Films and Characterization for Enhanced Biocompatibility
Source: Int J Biomater. 2022 Jul 31;2022:6331465. doi: 10.1155/2022/6331465 (PMC9467823; doi:10.1155/2022/6331465)
Supplement: Supplementary Materials — S1: SEM and TEM morphological characterizations of CaCO3 on the PVDF-HFP membrane and the corresponding EDS spectra with elemental composition. S2: performance characterization loading and release of a cationic dye (MB) into multilayers of (PAH/PAMAM)7.5-CaCO3 with different cross-linking degrees. S3: performance characterization loading and release of a cationic antibiotic (GS) into multilayers of (PAH/PAMAM)7.5-CaCO3 with different cross-linking degrees. [file 6331465.f1.docx]

# Supporting Information

**Layer-by-layer fabrication of PAH/PAMAM/nano-CaCO_3_ composite films and characterization for enhanced biocompatibility**

Naemi Tonateni. Shifeta ^1,2^, Shindume L. Hamukwaya ^2,3*^, An Qi ^2^, Huiying Hao ^2^, and Melvin Mununuri Mashingaidze^3^

^1^School of Science, University of Namibia, Windhoek 12010, Namibia.

^2^School of Materials Science and Technology, China University of Geosciences, Beijing 100083, People’s Republic of China.

^3^School of Engineering & the Built Environment, University of Namibia, Ongwediva 33004, Namibia.

E-mail: [nshifeta@unam.na](mailto:nshifeta@unam.na), [shamukwaya@unam.na](mailto:shamukwaya@unam.na), [an@cugb.edu.cn](mailto:an@cugb.edu.cn), [huiyinghaol@cugb.edu.cn](mailto:huiyinghaol@cugb.edu.cn), [mmashingaidze@unam.na](mailto:mmashingaidze@unam.na)

Correspondence should be addressed to; [shamukwaya@unam.na](mailto:shamukwaya@unam.na), (S.L. Hamukwaya)

## **1. SEM and TEM Morphological Characterizations of CaCO_3_ on the PVDF-HFP membrane**


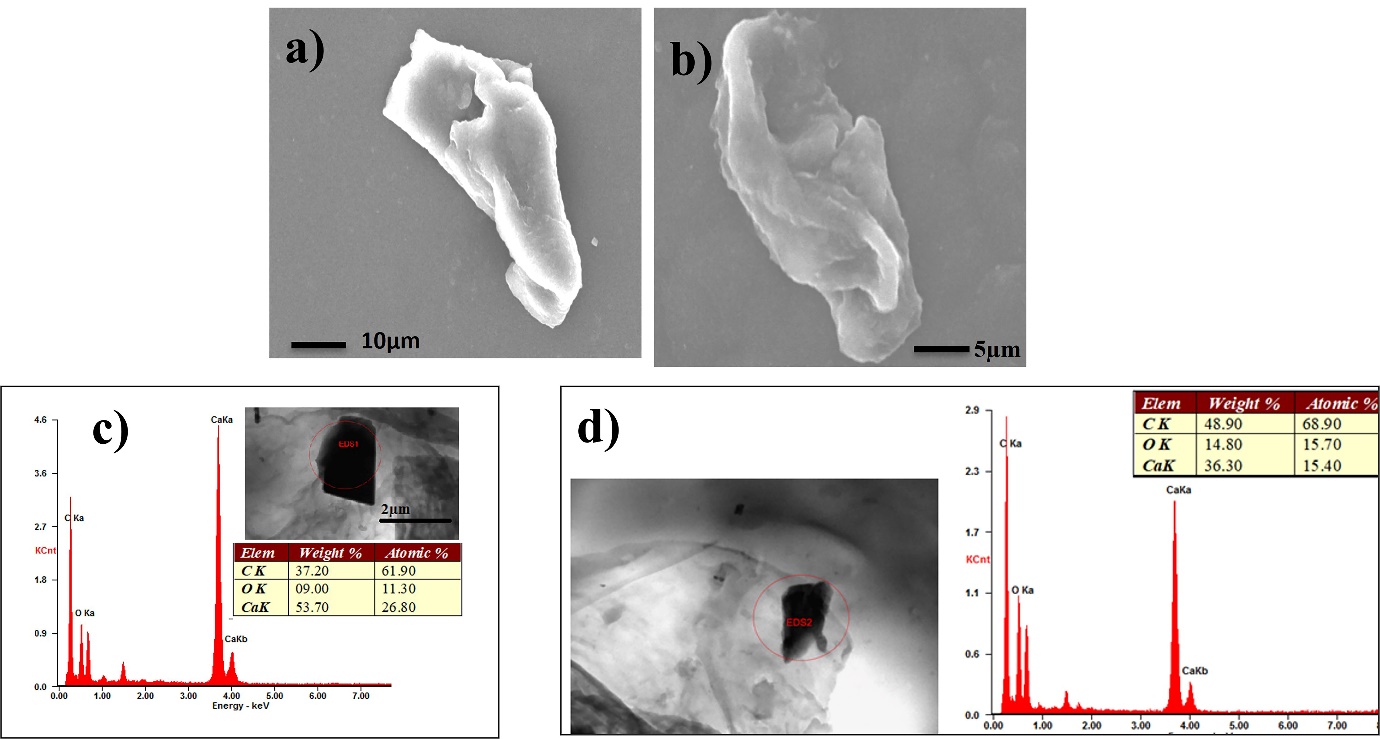


**S1**. Morphological Characterization of CaCO_3_ on the PVDF-HFP membrane. SEM images (a and b) of CaCO_3_ on the PVDF-HFP membrane on the medium cross-linked PAH/PAMAM)_7.5_ films. TEM images (c and d) of CaCO_3_ and their corresponding EDS spectra with elemental composition.

## **2. Performance Characterization- Loading and Release of a cationic dye (MB)**


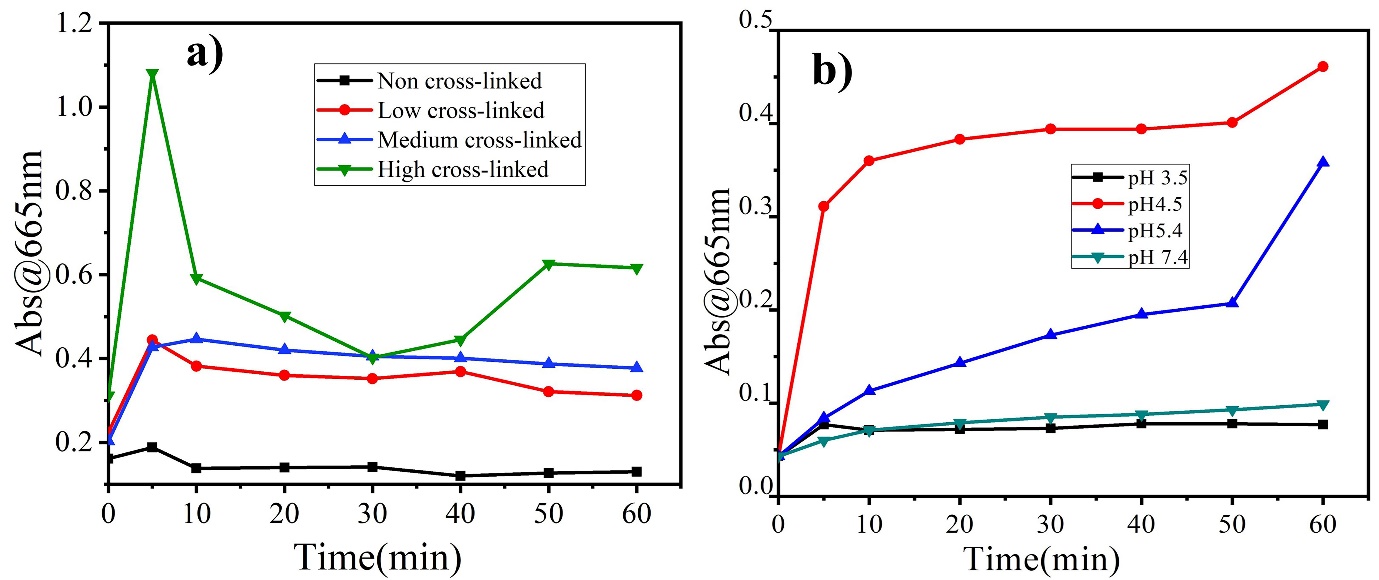


**S2.** Relationship of maximum absorbance (665nm) during the **a)** loading and **b)** release of MB (pH9) into multilayers of (PAH/PAMAM)_7.5_-CaCO_3_ with different cross-linking degrees.

## **3. Performance Characterization- Loading and Release of a cationic antibiotic (GS).**


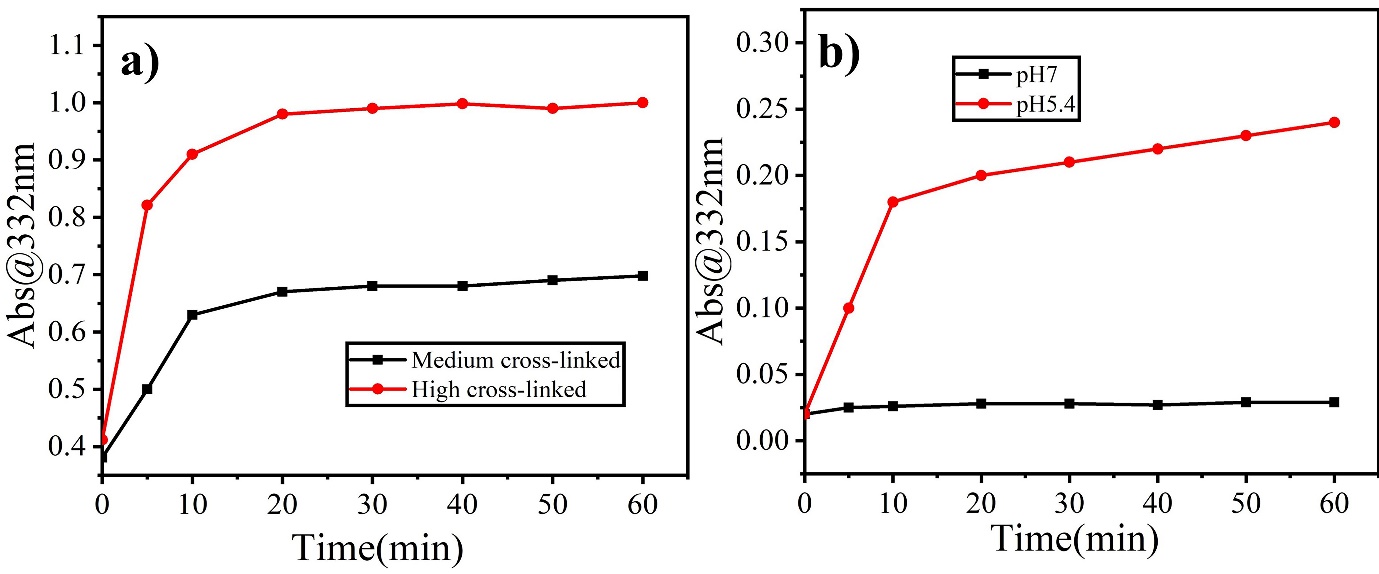


**S3.** Relationship of maximum absorbance (332nm) during the (a)loading and (b) release of Gentamycin sulfate (pH7) into multilayers of (PAH/PAMAM)_7.5_-CaCO_3_with ***different cross-linking degrees***.
